# An Uncommon Presentation of Erythrodermic Psoriasis in a Patient Without a History of Psoriasis

**DOI:** 10.7759/cureus.5099

**Published:** 2019-07-08

**Authors:** Matt Rendo, Joshua Boster, Scott R Dalton, Heather Yun

**Affiliations:** 1 Internal Medicine, Brooke Army Medical Center, Fort Sam Houston, USA; 2 Pathology and Dermatology, Brooke Army Medical Center, Fort Sam Houston, USA

**Keywords:** erythroderma, psoriasis, infliximab

## Abstract

Erythrodermic psoriasis is a rare and potentially fatal skin condition. We present the case of a 68-year-old woman, with no prior dermatologic history, who was treated with steroid injection for an insect bite one month prior to presentation and subsequently developed a diffuse erythematous rash. She presented to a military medical center in shock, with weeping, coalesced plaques covering the majority of her skin. Skin biopsies revealed pustular psoriasis, and treatment with infliximab was initiated for erythrodermic psoriasis. After six weeks and three infliximab infusions, the cutaneous lesions had nearly completely resolved.

## Introduction

Erythrodermic psoriasis (EP) is a rare, severe form of plaque psoriasis with the sudden development of inflammatory, erythematous skin plaques and edema. Diagnosis requires the involvement of over 75% of the total body surface area (TBSA) and may lead to metabolic and systemic compromise [1]. Untreated, the patient may succumb to multisystem organ failure and high output heart failure secondary to cutaneous volume loss [2]. We present a case of EP as a patient’s first presentation of psoriasis late in life.

## Case presentation

The patient is a 68-year-old morbidly obese female who developed a papulosquamous eruption under her breast one month after receiving a solumedrol injection for a presumed insect bite. Over one week, the rash spread to involve large areas of the trunk, proximal extremities, and scalp.

**Figure 1:**
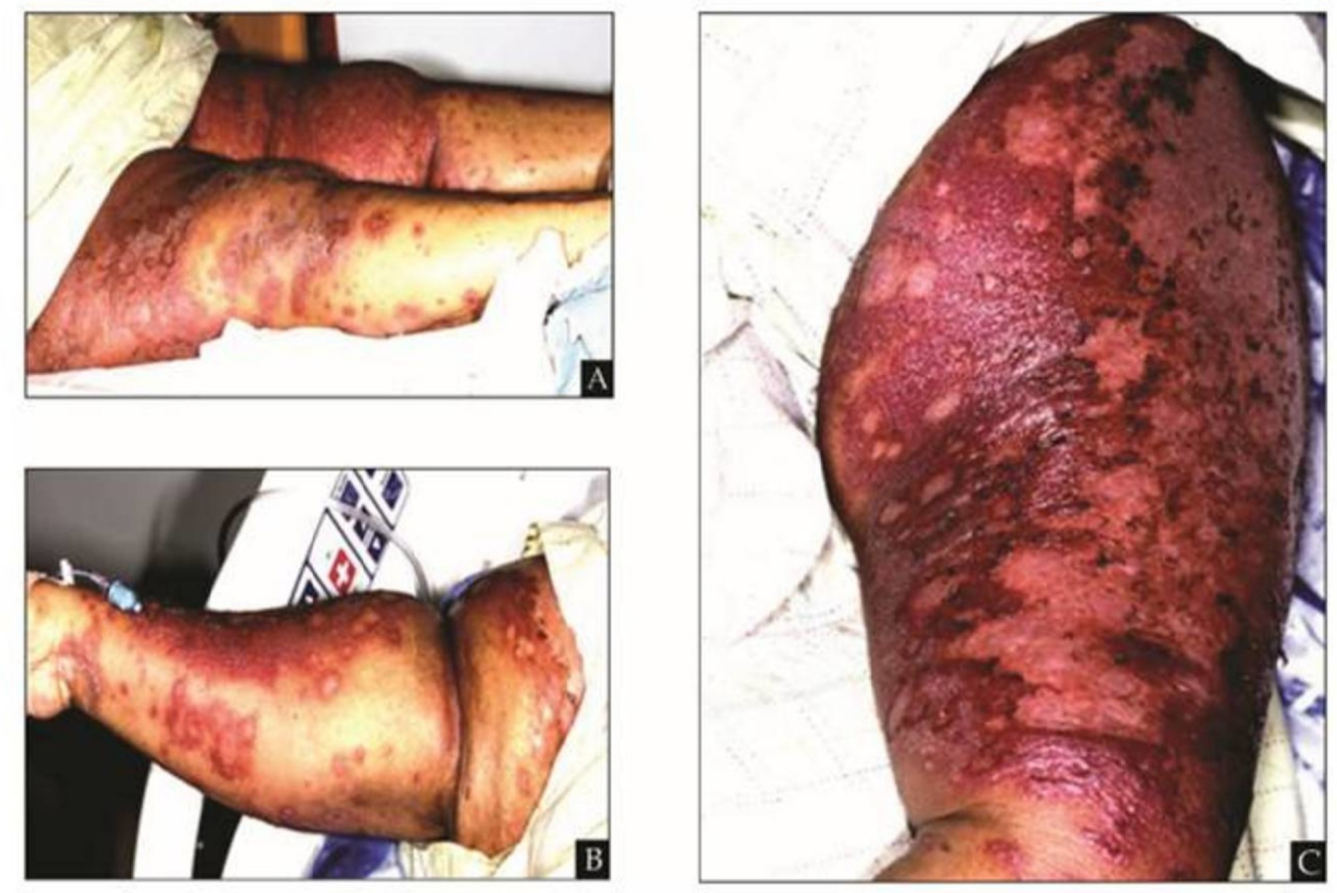
Bilateral proximal lower extremities with near complete plaque confluence with various sizes and states of plaque development on the distal lower extremities (A). Right forearm with scattered plaques coalescing on the lateral aspect of the forearm (B). Left forearm with near complete violaceous plaque confluence (C).

A skin biopsy performed in the clinic revealed psoriasiform dermatitis involving 40% of her TBSA. Topical triamcinolone cream was initiated. Two weeks later, she presented to the emergency room in shock with tachycardia, hypotension, diffuse skin pain, leukocytosis, and acute kidney injury. She had weeping, coalescing violaceous plaques and erosions on the chest, abdomen, back, and upper and lower extremities involving greater than 90% TBSA (Figure 1). There were pustules scattered across discrete plaques on the bilateral lower extremities. With no identifiable triggers or personal or family history of psoriasis, empiric antibiotic coverage with clindamycin and topical miconazole was initiated for a possible superinfection. A tuberculin skin test, chest X-ray, human immunodeficiency virus (HIV) test, and hepatitis panels were unremarkable. Dermatology was consulted, and she was continued on triamcinolone cream with wet skin wraps. Her acute kidney injury resolved with intravenous (IV) fluids, and she developed diffuse edema in the setting of continued weeping plaques. The pustules on her legs formed vesicles prior to rupturing with a gradual coalescence of the diffuse erythematous plaques.

**Figure 2:**
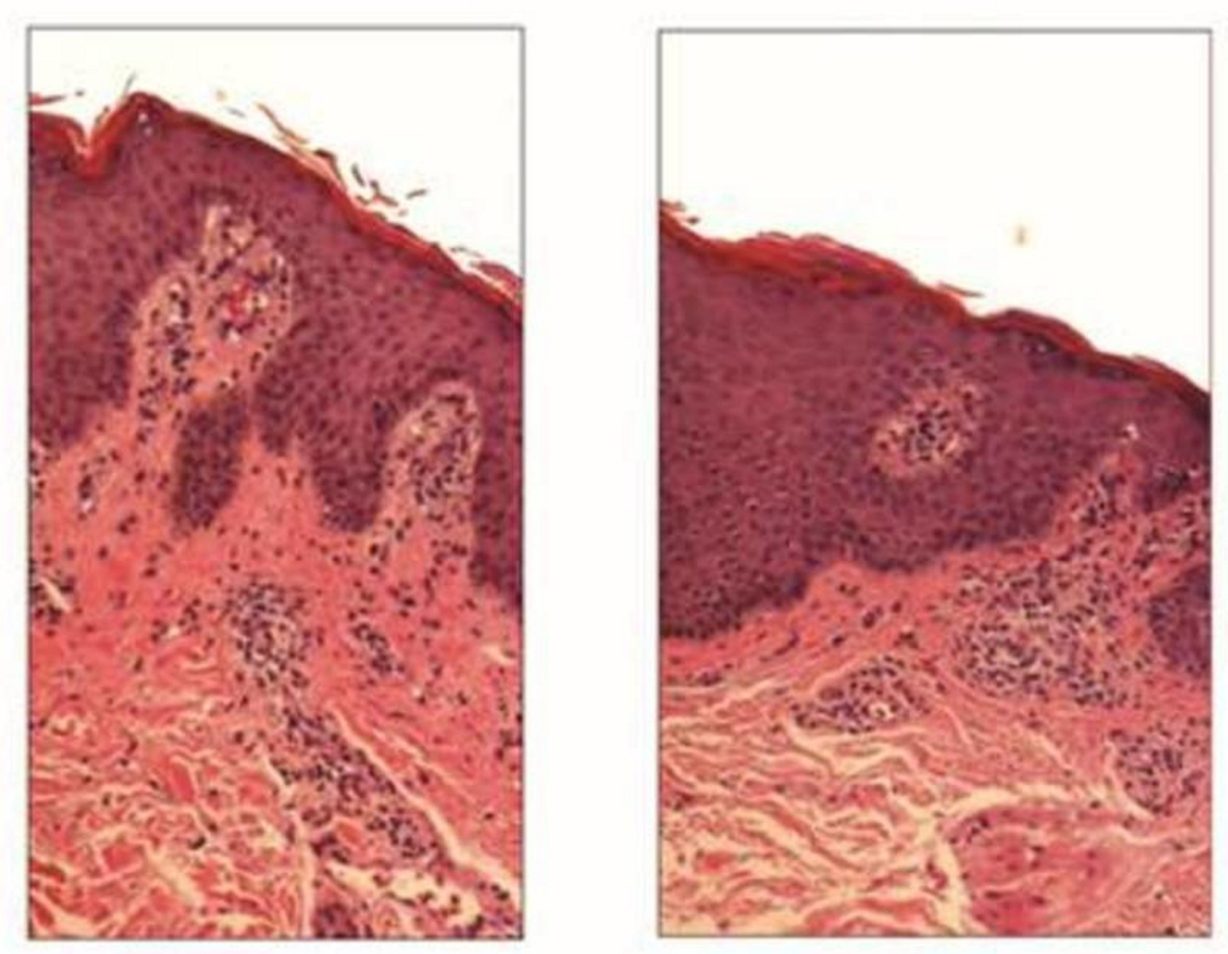
Skin biopsy illustrating psoriatic changes. Superficial perivascular lymphocytic infiltrate. The overlying epidermis shows regular psoriaform epidermal hyperplasia with thinning of the suprapapillary plates and dilated telangiectatic vessels in the papillary dermis. There is a loss of the granular cell layer in areas and neutrophils are present in the stratum corneum. Hematoxylin-eosin, original magnification x40.

Direct immunofluorescence was negative for immunoglobulin (Ig) G, IgA, IgM, and fibrin along the dermal-epidermal junction without evidence of IgA pemphigus. Repeat skin biopsy returned as pustular psoriasis (Figure 2). The patient was started on infliximab infusions. After the third infusion six weeks later, she had 10% TBSA still affected and was nearly lesion-free after five months.

## Discussion

EP is a rare and potentially fatal variant of plaque psoriasis believed to affect less than 3% of patients with psoriasis [3]. The majority of patients require hospitalization for fluid and electrolyte replacement in addition to assistance with wound care. Common etiologies for patients presenting with erythroderma include exacerbations of previous existing dermatosis, hypersensitivity drug reactions, and hematologic and cutaneous malignancies [4]. In 30% of cases, the etiology remains elusive. Furthermore, risk factors for the development of EP include a personal or family history of psoriasis, exposure to immunosuppressive medications, particularly systemic glucocorticoids, and HIV infection. With a broad differential diagnosis and no previous history of psoriasis, the diagnosis of EP in this patient was particularly challenging.

EP can be difficult to diagnose based on histopathology alone, and the clinical features often assist in determining the underlying cause. Notably, parakeratosis, acanthosis, and psoriasiform hyperplasia do not always indicate underlining psoriasis. This patient presented originally to an outpatient provider with concerns for an insect bite under her left breast described as a papulosquamous eruption. She received a steroid injection without clinical improvement. Subsequently, the rash rapidly progressed to cover over 90% of her body and prompted presentation to the emergency room. We presume that the initial insect bite was the first sign of psoriasis, which developed into EP in the setting of corticosteroid withdrawal. In this case, a correlation of subtle pathology and clinical history led to the diagnosis of EP. Treatment for EP includes adequate hydration, topical steroids and vitamin D analogs, management of associated infections, and immunosuppression for uncontrolled symptoms [5]. Cyclosporine is the drug of choice for EP. With cyclosporine’s association with nephrotoxicity, infliximab is often used in patients with renal disease or hypertension [5-7]. While there are no head-to-head trials comparing biologic therapy in patients with EP, over half are treated with infliximab [6]. After five months of treatment, this patient was nearly lesion free.

## Conclusions

This case represents an unusual presentation of EP in a patient without a prior history of psoriasis. When patients present critically ill with an erythrodermic rash, it is important to consider EP in the differential diagnosis, especially in the setting of a recent withdrawal of immunosuppression or steroid use. Skin biopsy and histological analysis aids in the diagnosis of EP given the broad differential associated with erythrodermic skin reactions. In this patient, treatment with infliximab resulted in rapid clinical improvement.
